# Finding the right fit: evaluation of short-read and long-read sequencing approaches to maximize the utility of clinical microbiome data

**DOI:** 10.1099/mgen.0.000794

**Published:** 2022-03-18

**Authors:** Jeanette L. Gehrig, Daniel M. Portik, Mark D. Driscoll, Eric Jackson, Shreyasee Chakraborty, Dawn Gratalo, Meredith Ashby, Ricardo Valladares

**Affiliations:** ^1^​ Siolta Therapeutics, 930 Brittan Ave, San Carlos, CA 94070, USA; ^2^​ Pacific Biosciences, 1305 O’Brien Dr, Menlo Park, CA 93025, USA; ^3^​ Shoreline Biome, 400 Farmington Ave, Farmington, CT 06032, USA

**Keywords:** microbiome, next-generation sequencing, long-read sequencing, live biotherapeutic product, 16S rRNA sequencing, metagenomics

## Abstract

A long-standing challenge in human microbiome research is achieving the taxonomic and functional resolution needed to generate testable hypotheses about the gut microbiota’s impact on health and disease. With a growing number of live microbial interventions in clinical development, this challenge is renewed by a need to understand the pharmacokinetics and pharmacodynamics of therapeutic candidates. While short-read sequencing of the bacterial 16S rRNA gene has been the standard for microbiota profiling, recent improvements in the fidelity of long-read sequencing underscores the need for a re-evaluation of the value of distinct microbiome-sequencing approaches. We leveraged samples from participants enrolled in a phase 1b clinical trial of a novel live biotherapeutic product to perform a comparative analysis of short-read and long-read amplicon and metagenomic sequencing approaches to assess their utility for generating clinical microbiome data. Across all methods, overall community taxonomic profiles were comparable and relationships between samples were conserved. Comparison of ubiquitous short-read 16S rRNA amplicon profiling to long-read profiling of the 16S-ITS-23S rRNA amplicon showed that only the latter provided strain-level community resolution and insight into novel taxa. All methods identified an active ingredient strain in treated study participants, though detection confidence was higher for long-read methods. Read coverage from both metagenomic methods provided evidence of active-ingredient strain replication in some treated participants. Compared to short-read metagenomics, approximately twice the proportion of long reads were assigned functional annotations. Finally, compositionally similar bacterial metagenome-assembled genomes (MAGs) were recovered from short-read and long-read metagenomic methods, although a greater number and more complete MAGs were recovered from long reads. Despite higher costs, both amplicon and metagenomic long-read approaches yielded added microbiome data value in the form of higher confidence taxonomic and functional resolution and improved recovery of microbial genomes compared to traditional short-read methodologies.

## Impact Statement

Accurate DNA sequencing and analysis approaches are essential for the development of microbiome-based therapeutics. The advent of high-fidelity (HiFi) long reads shifts the landscape of low-error microbiome sequencing options; however, a direct comparison of short- and HiFi long-read sequencing approaches has not been reported for clinical microbiome samples. Using stool samples collected from participants in a clinical trial of a live biotherapeutic product (LBP), we compare diverse microbiome data outputs generated from short-read and long-read metagenomic and 16S rRNA amplicon sequencing. While taxonomic profiles generally overlapped across methods, long-read sequencing provided higher taxonomic resolution and improved functional annotation. Detection of an investigational live biotherapeutic strain in clinical trial participants was achieved with all methods, and both metagenomic methods suggested active replication of this strain in treated trial participants. Metagenomic assemblies of short and long reads were compositionally similar, although long-read assemblies were more complete. We found that long-read approaches provide valuable additional information including greater taxonomic resolution with long-read amplicon sequencing and improved functional profiling and bacterial genome recovery from long-read metagenomics.

## Data Summary

All supporting data, code and protocols have been provided within the article or as supplementary data files. Five supplementary figures and six supplementary tables are available with the online version of this article. Sequencing data are accessible in the National Centre for Biotechnology Information (NCBI) database under BioProject accession number PRJNA754443. The R code and additional data files used for analysis and figure generation are accessible in a GitHub repository (https://github.com/jeanette-gehrig/Gehrig_et_al_sequencing_comparison).

## Introduction

The human microbiota consists of the trillions of micro-organisms colonizing the skin and mucosal surfaces. Much of the diversity and biomass of the microbiota resides in the intestinal lumen, with over 50 % of the solid fraction of stool consisting of bacterial cells [1]. The human gut microbiota harbours an immense amount of genetic material, known collectively as the microbiome. The gut microbiota performs essential functions for the human host, including supporting growth and development during infancy and childhood [2], educating the immune system [3, 4], protecting against pathogen colonization [5], synthesizing vitamins [6] and converting indigestible dietary components into usable energy sources [7]. Perturbations to the human microbiota are linked to a range of human conditions, from allergies and autoimmune diseases to cancer and autism [8, 9].

Advances in high-throughput DNA sequencing have expanded our understanding of the human microbiome and paved the way for the development of microbiome-based therapeutics. Live biotherapeutic products (LBPs) are a subcategory of microbiome-based interventions leveraged to treat, cure or prevent disease [10]. Bacterial LBPs may contain a single strain or a consortium of distinct strains, and may exert their effects directly through metabolic or immunologic effects or indirectly through impacting the composition and function of the microbiota [11]. Although LBPs may contain strains that are closely related to members of the native microbiota, distinct strains of the same bacterial species frequently display unique functional or structural characteristics with important implications for host–microbe interactions [12, 13]. Therefore, studies of microbiome-based interventions such as LBPs should aim to obtain strain-level resolution to accurately understand the impact of treatment on the taxonomic and functional capacity of the microbiota.

Methods to profile the human microbiome have evolved dramatically over the past three decades, with the effort and cost required for DNA sequencing falling precipitously. Nevertheless, metagenomic sequencing at the depth required to obtain comprehensive taxonomic and functional information from complex microbial communities remains relatively costly. Amplicon-based 16S ribosomal RNA (16S rRNA) gene sequencing remains the most common and economical sequencing approach for microbiome profiling. Comparison of the 16S rRNA gene sequence allows for an approximation of relatedness among taxa [14]. The 16S rRNA gene is approximately 1500 base pairs (bp) long and contains nine variable regions, which can be primed and amplified at lengths compatible with short-read Illumina DNA sequencing. Limited by read length, the choice of which variable region(s) to sequence has been a longstanding debate due to varying levels of amplification bias [15, 16]. Overall, short-read 16S rRNA gene sequencing generates community profiles with low taxonomic resolution that are difficult to interpret considering many bacterial genomes harbour multiple polymorphic copies of the 16S rRNA gene [17].

‘Third-generation’ long-read sequencing technology makes it possible to obtain full-length 16S rRNA gene sequences, eliminating bias in choosing a variable region while increasing taxonomic resolution. Historically high error rates (>10 %) per base of long-read sequencing limited its utility in microbiome profiling. Recently, Pacific Biosciences (PacBio) circular consensus sequencing (CCS) has significantly reduced errors by performing multiple passes of a circularized template molecule, generating HiFi reads, which are highly accurate (>Q20, median Q30-40) long reads over 10 kb in length [18]. CCS of the full-length bacterial 16S rRNA gene significantly improves taxonomic resolution in microbiome profiling, in many cases to the strain level. Sequencing a diverse (>250-member) mock community using HiFi sequencing can generate greater than 90 % accuracy in species-level classification with accurate measures of relative abundance [19]. Long reads are not limited to the 16S rRNA gene; amplicons can be extended to include the internal transcribed spacer (ITS) region and the 23S gene to provide even greater taxonomic resolution [20]. Regardless of the platform, 16S rRNA gene sequencing excludes non-bacterial/archaeal members of the microbiota, such as fungi, viruses, and eukaryotic cells, and does not provide direct information about the encoded functional capabilities of a microbiome [16].

As the microbiome field matures, a shift from descriptive observational studies to mechanistic studies is required to support the development of novel diagnostics and therapeutics [21]. The application of metagenomic sequencing for translational applications of microbiome profiling has expanded, partly based on improved detection of microbial species and encoded microbiome function [22]. While shallow shotgun sequencing (~0.5 million reads per sample) has been shown to capture the taxonomic and functional diversity of microbiome samples at relatively low cost, this approach does not provide sufficient coverage for *de novo* assembly of novel genes and genomes [23]. Since reference databases contain only a fraction of true microbial diversity, *de novo* assembly provides added value during microbial community profiling and strain discovery [24], a fact that has been highlighted by the recent additions of thousands of novel human gut microbiota strains through metagenomic assemblies [25, 26]. Even with deeper short-read shotgun sequencing, *de novo* assembly remains a challenge due to the presence of repetitive DNA regions, shared genomic regions between strains, and the complexity of microbial communities with unknown and uneven representation of both diverse and closely related strains [27]. Long-read sequencing can surmount many of the difficulties in metagenomic assembly by spanning repetitive sequences and highly conserved regions; however, reliance on short-read data to overcome high error rates and low coverage has made this approach costly and complex [28]. Notably, Oxford Nanopore sequencing is a low cost and convenient long-read sequencing approach that can be applied to microbiome profiling, particularly for fast pathogen identification, but higher error rates persist and require additional *in silico* computation or complementary short-read sequencing [29]. Though currently underexplored, the low error rates of HiFi reads could increase the value and utility of long-read sequencing for microbiome profiling.

The development and regulatory approval of microbiome-targeted therapeutics requires accurate approaches to obtain high resolution taxonomic and functional microbiome data. We applied four distinct microbiome sequencing approaches to stool DNA from participants enrolled in an LBP clinical trial to evaluate their utility for generating actionable data for clinical microbiome applications. Using amplicon-based sequencing, we compare the ubiquitous short-read V3-V4 16S rRNA amplicon sequencing (SRA) to long-read 16S-ITS-23S amplicon (LRA) sequencing. We also compare short-read metagenomics (Illumina, SRM) and long-read metagenomics (PacBio HiFi, LRM) outputs of the same clinical microbiome sample set. In addition, we determine whether an introduced therapeutic bacterial strain can be confidently detected during treatment using these distinct approaches. Finally, we compare the genome assemblies from the metagenomic sequencing methods, assessing the number, completeness and diversity of metagenome-assembled genomes (MAGs). As long-read sequencing incurs a greater per-sample sequencing cost, we evaluate sequencing method costs versus value of microbiome data obtained, focusing on two core areas of microbiome sequence data value: taxonomic resolution and encoded functional capacity.

## Methods

### Stool sample collection

After IRB approval and completion of informed consent, stool samples were collected from participants in STMC-103H-101, a clinical trial investigating the safety and tolerability of an LBP for the prevention of allergic disease. Participants were treated twice daily for 28 days with an investigational LBP or placebo, with clinical visits, which included faecal sample collection. Study subjects were provided with stool kits with collection containers (BioCollector, The Biocollective). Samples were frozen immediately after collection and remained frozen at ≤ −15 °C until processing.

A summary of the DNA extraction, library preparation, data analysis methods and databases used across the four sequencing and analysis methods is provided in Table S1 (available in the online version of this article) and detailed below.

### SRA (V3-V4 16S rRNA) sequencing and analysis

Stool sample DNA was extracted using the CTAB method as previously described [30]. DNA concentrations were measured using the Qubit dsDNA Broad Range Assay Kit on the Qubit 4 Fluorometer (ThermoFisher Scientific), and DNA quality was assessed using the 260/280 and 260/230 ratios measured using a NanoDrop Spectrophotometer.

DNA samples were normalized to 10 ng µl^−1^ in TE buffer. Dual-indexed libraries were prepared following Illumina’s 16S Metagenomic Sequencing Library Preparation Protocol for the MiSeq System (Part No. 15 044 223 Rev. B). Briefly, the variable V3 and V4 regions of the 16S rRNA gene were amplified using the following primers with Illumina overhang adapter sequences: 16S Amplicon PCR Forward Primer=5′ TCGTCGGCAGCGTCAGATGTGTATAAGAGACAGCCTACGGGNGGCWGCAG. 16S Amplicon PCR Reverse Primer=5′ GTCTCGTGGGCTCGGAGATGTGTATAAGAGACAGGACTACHVGGGTATCTAATCC. PCR amplicons were purified using magnetic beads (Omega Bio-tek), and each sample was uniquely indexed using Nextera XT Index Kit (Illumina, Cat. No. FC-131–2001). Indexed libraries were quantified using the Qubit dsDNA High Sensitivity Kit (ThermoFisher Scientific). Amplicon libraries were pooled equally by DNA concentration and sequenced on a 2×300 bp MiSeq run.

Demultiplexed fastq files were imported into QIIME 2 for sequence processing and analysis [31]. Reads were quality filtered, merged, denoised and chimaeras removed using DADA2 [32] in QIIME 2 (*qiime dada2 denoise-paired*; adjusted parameters: --p-max-ee-f 4, --p-max-ee-r 4, --p-trim-left-f 17, --p-trim-left-r 21, --p-trunc-len-f 268, --p-trunc-len-r 214). The resulting table contained the number of reads in each sample assigned to amplicon sequence variants (ASVs). ASVs were assigned taxonomy using naive Bayes classifiers trained on Greengenes 13_8 99 % OTUs [33] or silva version 132 99 % OTUs [34] extracted for the target V3-V4 region. ASVs with fewer than 10 reads across all samples were removed. To detect the active ingredient strain, DSM 33213, the number of reads in each sample assigned to DSM 33213’s 408-bp-long V3-V4 ASV were counted and divided by the total number of reads per sample.

### LRA (16S-ITS-23S) sequencing and analysis

To compare the impact of DNA extraction method on microbiome profiles, DNA was extracted from aliquots of each sample using the AllPrep PowerFecal DNA/RNA kit (Qiagen), the CTAB buffer-based protocol [30], and Shoreline Complete DNA extraction (StrainID kit, Shoreline Biome). Each sample was prepared in duplicate using the StrainID kit, and 10 ng of DNA from each sample extracted using the AllPrep PowerFecal kit and CTAB buffer-based DNA extraction were added to the StrainID plate prior to amplification. In addition to faecal samples, both DNA (10 ng) and cells (10^8^ and 10^9^) from strains were included in the StrainID library preparation as references. The StrainID amplicon spans the full 16S and partial 23S rRNA genes by PCR amplification using target specific primer pair pools forward 5′-AGRRTTYGATYHTDGYTYAG and reverse 5′-AGTACYRHRARGGAANGR. LRA library preparation, sequencing and analysis were performed as previously described [20].

SBanalyzer 3.0 was used to demultiplex and classify CCS fastq reads from the Sequel II sequencing run with the StrainID_PacBio_species pipeline, using the Athena database for taxonomic classification. The untrimmed concatenated fastq files were processed using DADA2 in R as previously described [20]. Singleton sequences were removed from consideration during inference of ASVs (the dada2 parameter DETECT_SINGLETONS was not set to TRUE). This means that only sequences occurring two or more times in the pooled dataset were candidate ASVs, which is default behaviour.

*PICRUSt2* analysis was performed to predict metagenome function from amplicon sequencing data. Reads were clustered and polished using a reimplementation of the NanoCLUST pipeline [35] to generate the representative sequences and associated per-sample abundances. The python script picrust2_pipeline.py from the PICRUSt2 distribution was applied [36]. Since the 16S-ITS-23S sequences are much longer than the 16S sequences in the PICRUSt2 database, a minimum alignment length threshold of 0.5 was used (default for --min_align is 0.8). There were 65 of 3060 sequences that did not meet that threshold and were filtered out. The flags --stratified and --per_sequence_contrib were used.

### SRM (short-read metagenomics) sequencing and analysis

Stool sample DNA was extracted using the CTAB method described above. Libraries were prepared using scaled-down tagmentation Nextera XT DNA Library Prep Kit (Illumina) protocol [37]. Libraries were quantified using the Qubit 1 x dsDNA High Sensitivity Kit (ThermoFisher Scientific) and pooled equally by DNA concentration. The final pool was sequenced on one lane of a NovaSeq 6000 S4 150 bp PE flow cell (Illumina).

Nextera adapter sequences were trimmed from demultiplexed paired reads using bbduk (BBTools, sourceforge.net/projects/bbmap/) and reads 105 bp and longer were retained. Reads mapping to the human genome (GRCh38) were removed using bbsplit (BBTools, sourceforge.net/projects/bbmap/). Kraken2 was used to assign taxonomy to reads [38] and Bracken was used to estimate species abundance [39]. HUMAnN2 was used for functional annotation, using the UniRef90 protein database [40]. Clean reads were mapped to the investigational product strain using bbmap (BBTools, sourceforge.net/projects/bbmap/) in perfect mode, meaning counted reads must map to the reference perfectly without substitutions or indels. The fraction of R1 reads mapping to the reference genome was counted as the mapped read fraction.

Clean reads were assembled using metaSPAdes in paired-read mode with default settings [41]. For metagenome assembly evaluation and MAG identification, the HiFi-MAG-Pipeline was used (https://github.com/PacificBiosciences/pb-metagenomics-tools). A brief overview of this workflow is described in the following section on LRM Sequencing and Analysis. The short-read analysis required several modifications. For each sample, a reads file consisting of the pre-processed, interleaved R1 and R2 files was input for coverage calculations. The command used to map reads to contigs was changed to accommodate short reads: minimap2 -axe sr. The filtering parameters for the maximum number of contigs allowed in a bin was changed from 20 to 500.

### LRM (long-read metagenomics) sequencing and analysis

DNA was extracted from aliquots of each sample using the AllPrep PowerFecal DNA/RNA kit (Qiagen) to obtain high molecular weight DNA required for LRM. DNA fragment lengths were measured using TapeStation 4200 (Agilent). DNA fragment average lengths were approximately 8–10 kb, so no additional shearing was required. Sequencing libraries for each sample were created using the SMRTbell Express Template Prep Kit 2.0 (PacBio) per manufacturer’s instructions. Libraries were sequenced with four samples per run on a Sequel II System (PacBio). One library failed sequencing due to a problem with its library preparation (treatment sample from participant 6). After sequencing, CCS analyses were run using SMRTLink software v10 to produce HiFi reads for each sample.

Taxonomic and functional profiling was performed using a PacBio pipeline (Taxonomic-Functional-Profiling-Protein; https://github.com/PacificBiosciences/pb-metagenomics-tools). This pipeline uses DIAMOND [42] to perform translational alignment of HiFi reads to a protein database (e.g., NCBI nr). The resulting alignments are interpreted using MEGAN-LR [43, 44], which uses a lowest common ancestor (LCA) algorithm and other long-read settings to assign taxonomic and functional annotations to the HiFi reads. One particularly important setting for MEGAN-LR is the minimum support (as a percent of assigned reads) required to make a taxonomic assignment (--minSupportPercent). This setting was recently optimized in a benchmarking study using mock community datasets [45], allowing high-precision detection of species at 0.1 % abundance using a setting of 0.01 (versus the default of 0.05). Due to abundance designs of the mock communities, this study was unable to determine the exact lower limit for detection using this setting, but indicated the range is between 0.02 and 0.1 %. The outputs include absolute and normalized taxonomic read counts using a taxonomy from NCBI (NCBI nr database downloaded in August 2020) or the Genome Taxonomy Database (release 95) [46, 47], as well as counts for functional annotations based on InterPro2GO, SEED, EC and eggNOG [43].

LRM reads were aligned to the DSM 33213 reference genome using minimap2 with the most sensitive settings possible (resembling blastn). Alignments were filtered to keep hits representing primary alignments with >5000 matched bases and >98 % identity to the references. The number of hits per reference strain was normalized based on the number of total reads.

Metagenomic assemblies were performed for each HiFi read set with hifiasm-meta [48] using the default settings. To evaluate the assemblies and identify high-quality MAGs, the PacBio HiFi-MAG-Pipeline was used (https://github.com/PacificBiosciences/pb-metagenomics-tools). Briefly, HiFi reads were aligned to contigs to obtain coverage estimates, which were used with MetaBat2 [49] to perform binning. A separate bin set was also constructed from all circular contigs (one bin per circular contig), and the two binning strategies were compared and merged using DAS_Tool [50]. Resulting bins were evaluated using CheckM [51], and quality thresholds were applied to retain high-quality MAGs (>70 % completeness, <10 % contamination, <20 contigs). High-quality MAGs were analysed using the Genome Taxonomy Database Toolkit (GTDB-Tk) [52], which identified the closest reference genome for taxonomic assignment.

### Target strain matching for metagenomic contigs

The reference genome of the investigational product active ingredient strain DSM 33213 was mapped to assembled contigs using minimap2 [53]. Contigs belonging to the active ingredient strains were identified by creating scatterplots of the resulting alignment lengths and number of matched bases. In the scatterplot, perfectly matched contigs (to a strain) occurred on a line with a slope (*m*) of 1 and intercept (*b*) of 0, indicating the number of matched bases was equal to the alignment length (100 % identity).

### Comparing taxonomic profiles from different sequencing methods

The pcoa.plot function from the RAM package (version 1.2.0) in R was used to generate principal coordinates analysis (PCoA) plots of Bray–Curtis distances between samples using the taxonomy relative abundance tables at the lowest available taxonomy level for each sequencing method. Pairwise Mantel tests were performed in R using the mantel.rtest from the ade4 package (version 1.7–16) with 9999 permutations.

### Mapping SRA reads to LRA reads

For each sample, the LRA reads (from the replicate with the most reads that went through the entire StrainID protocol) were mapped to the same sample’s unique ASV sequences using minimap2 to generate paf alignment files. Perfect alignments with greater than or equal to 400 matching base pairs were considered hits.

### Number of unique taxa detected across methods

For SRA, the number of unique taxa was defined as the number of unique ASVs present in each sample from the filtered ASV table. ASVs were filtered out if they had fewer than 10 reads across all samples. For LRA, the number of unique taxa per sample was determined by counting the taxa present at taxonomy level 8 (strain level) from the Athena database assignments. For SRM, taxa assigned by Kraken2 and normalized at the species level by Bracken that were present at greater than or equal to 0.05 % relative abundance were counted. This was the threshold below which reads in a mock community were assigned to taxa not present in the mock community. For LRM, read counts were obtained for taxa using MEGAN-LR. The number of taxa assigned to the species-level with NCBI taxonomy were counted.

### Taxonomic resolution

For SRA, reads were considered to have species-level taxonomy if the Greengenes taxonomy classifier (or silva taxonomy classifier for silva analysis) assigned the ASV to a named species (i.e. not unknown or unclassified). For LRA, the replicate of each sample that underwent the entire StrainID protocol and had the greatest number of reads was chosen to assess taxonomic resolution. Reads assigned to taxonomy level 7 with a specific named species (i.e. not unclassified) were counted as having species-level resolution. Reads assigned to taxonomy level 8 with a specific named strain (i.e. not unclassified) were counted as having strain-level resolution. The denominator was the total number of reads assigned to ‘Bacteria’.

### Differential abundance

The R package MaAsLin 2 (Microbiome Multivariable Associations with Linear Models) [54] was used to identify differentially abundant bacterial families and differentially abundant functional pathways with time point as a fixed effect and study participant as a random effect. For LRM, taxonomy assignments based on the NCBI were used. Family and pathway abundances were normalized with total sum scaling and log transformed.

### Statistics

Statistical analyses were performed in R (version 4.0.4) and Prism 9 (version 9.1.2), as detailed for each analysis.

## Results

### Summary of sequencing and bioinformatic approaches

**Fig. 1.**
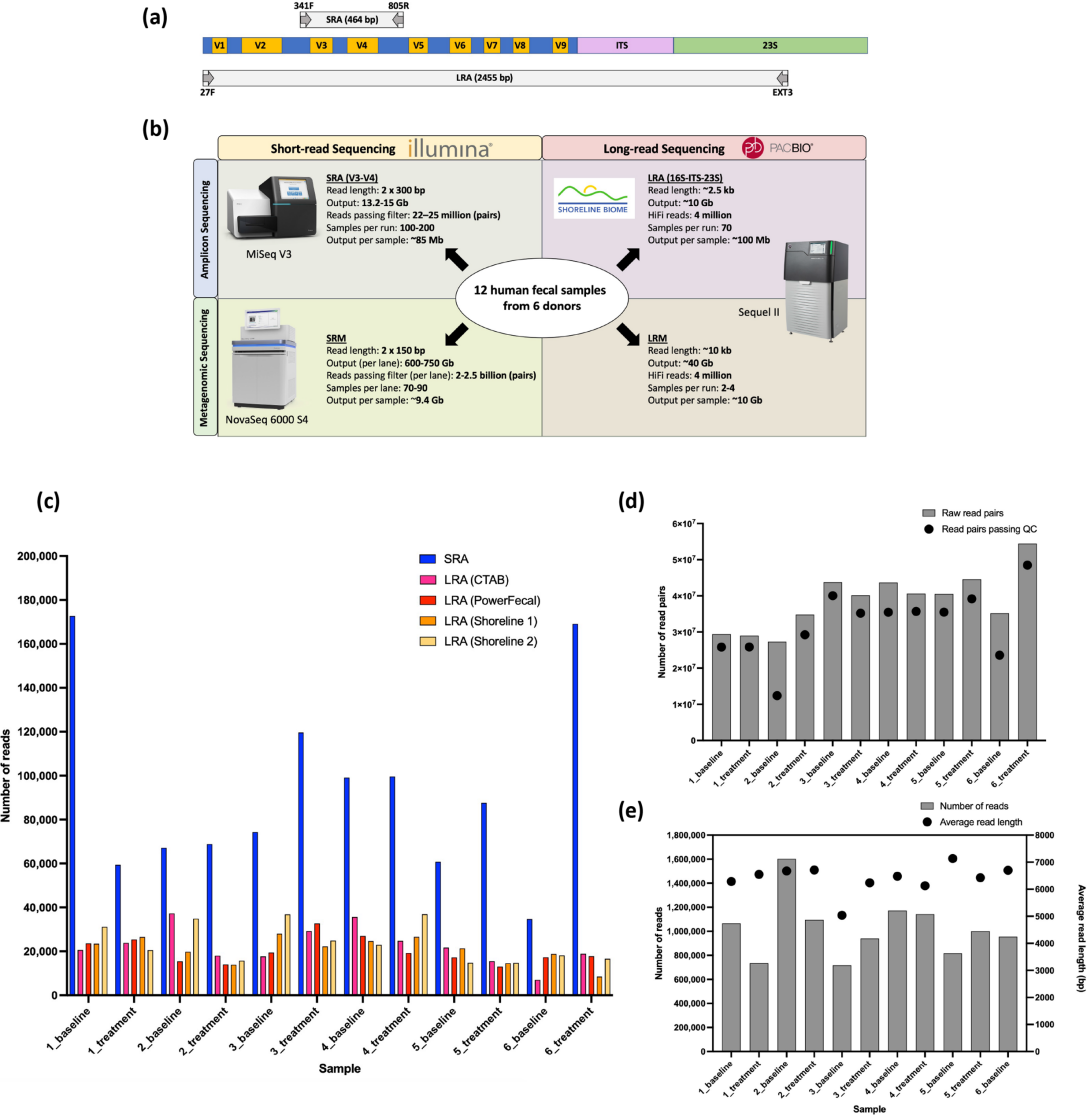
Summary of sequencing methods and output data. (a) Comparison of SRA and LRA amplicons. SRA consists of a portion of the 16S rRNA gene spanning variable regions 3 and 4. LRA spans the entire 16S rRNA gene, the ITS region, and part of the 23S rRNA gene. (b) Summary of the four sequencing methods compared in this analysis, including the sequencing platforms, read lengths, data outputs and multiplexing capabilities. (c) Number of reads passing demultiplexing and filtering for SRA and the number of HiFi reads passing demultiplexing and classification for LRA by sample replicate. For LRA, the DNA extraction method used for each replicate is denoted in parentheses in the legend. (d) The number of raw read pairs and the number of read pairs passing filtering for SRM. (e) The number of LRM reads and the average read length per sample. Participant 6's treatment sample failed LRM library preparation and was excluded from analysis.

We applied the 515F and 803R V3-V4 primer pair for short-read amplicon (SRA) library preparation in this analysis because it represents the most common historic amplicon approach for microbiome analyses, and was shown to be optimal for profiling bacterial communities [55] and maximizing phylogenetic coverage [56]. Long-read amplicons (LRAs) included the full-length 16S rRNA gene, the ITS region and part of the 23S gene (Fig. 1a). SRA and short-read metagenomic (SRM) libraries were sequenced 2×300 bp on an Illumina MiSeq and 2×150 bp on an Illumina NovSeq, respectively. Long-read metagenomic (LRM) and LRA libraries were sequenced on the PacBio Sequel II System. A summary of the four sequencing methods compared in this analysis is provided in Table 1.

**Table 1.** Summary of sequencing methods for microbiome profiling

Method	Sequencing type	Sequencing platform	Read lengths (bp)	No. of reads per sample (M)	Output	Data processing required	Cost per sample
SRA (V3-V4)	Amplicon	Illumina MiSeq	250 or 300	0.03–0.2	Taxonomic profiling (genus level)	Low	$
LRA (16S-ITS-23S)	Amplicon	PacBio Sequel II	~2500	0.01–0.03	Taxonomic profiling (strain or species level)	Low	$
SRM (short-read metagenomics)	Metagenome	Illumina NovaSeq 6000	150	20–100	Taxonomic and functional profiling, genome assembly and binning	High	$$
LRM (long-read metagenomics)	Metagenome	PacBio Sequel II	7000 to>10 000	1.5–3	Taxonomic and functional profiling, genome assembly and binning	Moderate	$$$

Total data output per sample was comparable across the two amplicon sequencing methods and the two metagenomic sequencing methods, with both metagenomic sequencing methods resulting in about 100 times the data of the amplicon sequencing methods (Fig. 1b). Notably, the per-sample costs of SRA and LRA are similar, while the per-sample cost of LRM is substantially higher than SRM (Table 1). This is due, in part, to the lack of high-throughput metagenomic sequencing on the Sequel II System; this experiment included four samples per sequencing run.

### Sample and data summary

The faecal samples analysed for this comparison were collected from subjects at two timepoints: pre-treatment baseline and 4 weeks after investigational LBP treatment. Twelve faecal samples from six clinical trial participants were sequenced using four methods: SRA, LRA, SRM and LRM (Fig. 1b). The SRA libraries sequenced on the Illumina MiSeq resulted in an average of 92 766 (±42 804 sd) combined read pairs per sample after denoising (Fig. 1c, Methods). LRA libraries sequenced on the Sequel II resulted in an average of 21 852 (±7307 sd) HiFi reads per sample (Fig. 1c). SRM libraries sequenced on the Illumina NovaSeq 6000 resulted in an average of 38.6 (±7.8 SD) million 150 bp read pairs per sample; on average, 82.2 % (±13.2 % sd) of the raw reads passed filtering criteria (Fig. 1d, Methods). LRM libraries sequenced on the Sequel II System produced an average of 1.0 million (±0.25 sd) reads per sample, with average read lengths per sample ranging from 5 to 7.1 kb (Fig. 1e).

### Comparing taxonomic profiles across methods

**Fig. 2.**
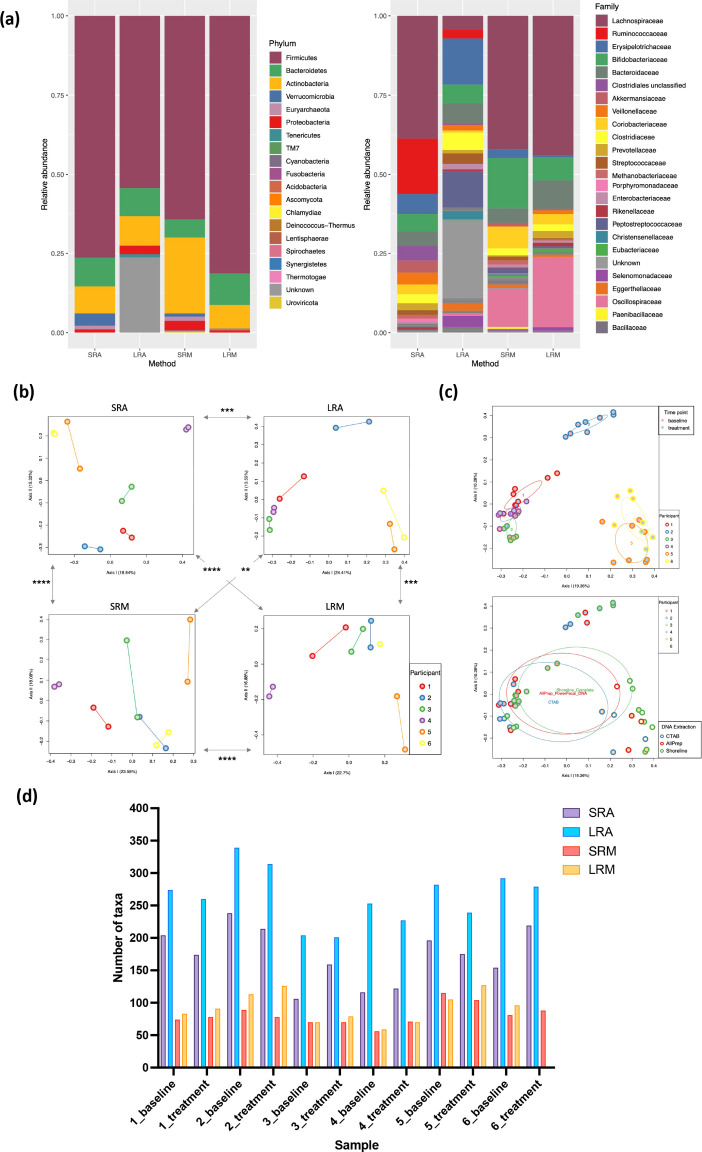
Taxonomic comparisons across methods. (a) Taxonomic comparisons show the relative abundance of phyla or families across all samples. All phyla and the 25 most abundant families across all methods are shown. (b) Bray–Curtis distances between samples were calculated using the lowest taxonomic level available for each method (species, strain, ASV). Samples are coloured by participant with samples from the same participant connected by a straight line. Pairwise Mantel tests were performed between Bray–Curtis distance matrices, and significance based on simulated *P*-values for each comparison are shown with arrows indicating the two distance matrices compared (*P <0.05; **P <0.01; ***P <0.001; ****P <0.0001). (c) Bray–Curtis distances for LRA replicates are shown on PCoA plots. Left plot samples are coloured by participant and sample time point, with ellipses for samples from the same participant. There was significant clustering by participant. Right plot samples are coloured by DNA extraction method and participant, with ellipses for DNA extraction method. There was no significant clustering by DNA extraction method. (d) The number of unique taxa detected for each sample, including unknown taxa, at the lowest level of taxonomy for each method.

To determine if short-read and long-read approaches generated comparable taxonomic profiles at higher-order taxonomies, all samples were combined and the relative abundances collapsed at phylum and family levels (Fig. 2a). Firmicutes was the dominant phylum across all methods (average abundances of 76, 54, 64 and 81%, for SRA, LRA, SRM and LRM, respectively) followed by Actinobacteria and Bacteroidetes. The overall proportions of phyla were similar across methods, with some notable differences. Unlike other methods, LRA resulted in a significant proportion of unknown phyla (24%). Most of the reads that mapped to ‘unknown phyla’ (22.8 %) were classified at the species level as ‘bacterium LF-3’. Since bacterium LF-3 is not classified into higher-order taxonomies, the reads that mapped to this bacterium remained unclassified at all other levels. SRM resulted in a greater proportion of Actinobacteria compared to the other methods (24 % for SRM compared to 8.5 % for SRA, 9.3 % for LRA, and 7.4 % for LRM; one-way ANOVA *P*<0.0001). SRA and SRM identified a significantly greater proportion of Verrucomicrobia (3.9 % for SRA and 1.0 % for SRM compared to 0.16 % for LRA and 0.28 % for LRM; one-way ANOVA *P*=0.007). SRA, SRM and LRM also identified the Archaea phylum Euryarchaeota (1.2 % for SRA, 1.3 % for SRM and 0.28 % for LRM), which was not identified with LRA.

Comparing relative abundances at the lower taxonomic level of family shows broad similarities with a few notable differences. Lachnospiraceae was the dominant family across all methods except for LRA, which had a greater proportion of unknown families (Lachnospiraceae abundance 39 % for SRA, 4.3 % for LRA, 42 % for SRM, and 44 % for LRM). LRA also had a significantly greater proportion of the family Peptostreptococcaceae (12 % for LRA compared to 0.33 % for SRA, 1.6 % for SRM, and 0.53 % for LRM; one-way ANOVA *P*<0.0001). Both amplicon methods resulted in a higher relative abundance of the family Erysipelotrichaceae (6.3 % for SRA and 15 % for LRA compared to 2.8 % for SRM and 5.8 % for LRM; one-way ANOVA *P*=0.0006). Notably, the family Ruminococcaceae was missing from both metagenomics methods. This is likely due to differences in taxonomy databases, because when an older version of NCBI or GTDB were used to assign taxonomy to SRM and LRM, respectively, Ruminococcaceae was an abundant family with both methods (Fig. S1). When a more recent version of NCBI was used to assign the taxonomy shown in Fig. 2(a), the reads originally assigned to the family Ruminococcaceae were assigned to Oscillospiraceae.

To directly assess the overlap of the inferred microbial communities from the amplicon-sequencing methods without relying on reference databases, we mapped each sample’s LRA reads to that sample’s unique SRA ASVs (Methods, Table S2). Across samples, an average of 29 % (±9 % sd) of each sample’s unique SRA ASVs above 0.05 % relative abundance mapped to the sample’s LRA reads. The ASVs with perfect mappings constituted an average of 24 % (±14 % sd) total community abundance. Conversely, an average of 13 % (±4 % sd) of each sample’s LRA reads perfectly mapped to SRA ASVs. The taxonomy assigned to the mapped ASVs largely overlapped with the taxonomy assigned to the LRA reads. For example, for participant 1’s baseline sample, 82 % of the overlapping reads had the same taxonomy (Table S2b).

To compare sample taxonomic profiles across methods using the highest taxonomic resolution available to each method, we calculated Bray–Curtis distances (which factors presence/absence and relative abundance of taxa) between samples at the lowest taxonomic level for each method [species, strain, or amplicon sequence variant (ASV)], and compared the distances between samples across methods. Notably, principal coordinates analysis (PCoA) plots of Bray–Curtis distances show that independent of the sequencing method, samples cluster by individual (Fig. 2b). Pairwise Mantel tests were performed between Bray–Curtis distance matrices from the four methods and all matrices were significantly positively correlated, suggesting that all four methods preserved relationships between the samples (Fig. 2b). Across LRA replicates, samples significantly clustered by participant (PERMANOVA *P*=0.001) but not by DNA extraction method (PERMANOVA *P*=0.2) (Fig. 2c), showing that the DNA extraction methods used did not drastically impact the overall taxonomic profiles.

### Identification of unique taxa across methods

Microbiome profiling approaches should maximize the identification of unique bacterial taxa while minimizing artefacts from DNA library preparation and sequencing. For the amplicon approaches, taxa were counted at the ASV or strain level (even if the ASV or strain had unknown taxonomy), while for metagenomic methods, the number of unique species were counted. After filtering out noise from SRM, amplicon approaches detected more unique taxa per sample than metagenomics (Fig. 2d). For SRM, the average number of species with >0.01 % abundance is 426 (±112 sd), with an average of 264 (61 %) of those species occurring in the 0.01–0.05% abundance zone. By contrast, for LRM only a limited number of taxa were detected between 0.01–0.05% abundance (Table S3). Closer inspection revealed the lower limit for detection was 0.04 % relative abundance, which is within the lower limit range of 0.02–0.1% reported by Portik *et al*. [45]. To facilitate comparisons between the SRM and LRM methods, we only compared taxa above 0.05 % relative abundance. Numbers were similar between methods across higher abundance zones, with LRM detecting slightly more taxa on average than SRM across the 0.05–0.1 %(28 versus 26), 0.1–1 % (46 versus 40), and >1 % levels (18 versus 15). The total number of species with >0.05 % abundance was 81.2 (±16.0 sd) for SRM and 93 (±23.0 sd) for LRM, indicating LRM had potentially higher detection ability in this zone relative to SRM.

**Fig. 3.**
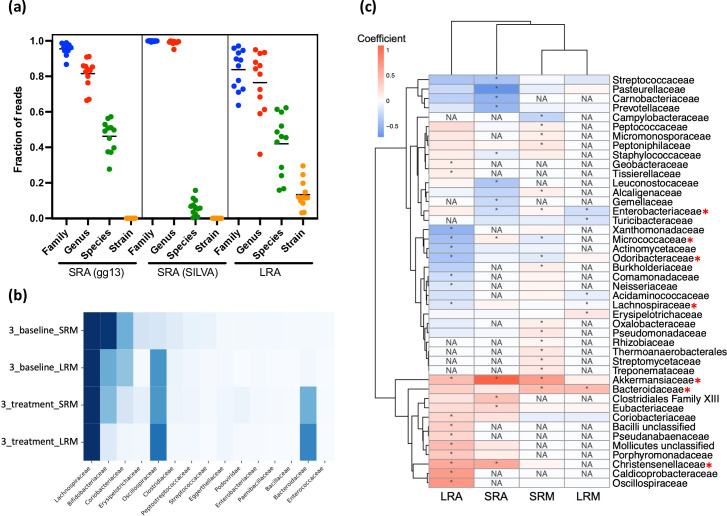
(a) The fraction of reads for each sample assigned to the specified taxonomy level for SRA and LRA. Horizontal lines show the mean fraction of reads assigned to each taxonomy level for each method. (b) Comparison of family relative abundances in participant 3’s baseline and treatment samples across metagenomics methods. The scale shows percent relative abundance. (c) The heatmap shows the families that were significantly differentially abundant between baseline and treatment samples in at least one method (asterisks in heat map denote *P*<0.2 for SRA, LRA, and SRM and *P*<0.25 for LRM). The red asterisks indicate families that were differentially abundant in more than one method.

No significant correlations were observed between the number of reads and the number of unique taxa detected, suggesting that sequencing depth was sufficient to capture stool bacterial diversity (Fig. S2). There was no significant difference between the percent of reads assigned to species for SRA with Greengenes taxonomic assignment (46.2±8.9 % sd) and LRA (42.0±16.8 % sd)(Fig. 3a) . However, the percent of species-level assignments was significantly greater with LRA compared to SRA with silva taxonomy assignments (6.3±4.5 % sd), highlighting the major impact of reference database selection. Only reads assigned a named species from the specified taxonomy database (Greengenes or silva for SRA, Athena for LRA) were considered to have species-level taxonomic assignments. In addition to species-level assignments, 13.4 % (±7.9 % sd) of LRA reads were assigned a strain designation(Fig. 3a).

Comparing the taxonomic assignments of the unique SRA ASVs and the LRA reads to which they mapped allowed for a direct comparison of taxonomic resolution across amplicon sequencing methods. For each sample, we considered the reads that mapped perfectly between methods. For these overlapping reads, the two methods displayed comparable genus-level resolution of 71 % for SRA (Greengenes taxonomy) and 64 % for LRA. However, an average of 30 % of overlapping reads were assigned to a known species for SRA, while 51 % were assigned a known species for LRA. Moreover, 11 % of the LRA reads with perfect alignments to ASVs were assigned a strain designation, while strain-level resolution is not possible for SRA sequencing (Table S2c). Interestingly, 30 % of LRA reads that mapped to SRA ASVs were assigned the taxonomy ‘bacterium LF-3’. Twelve unique SRA ASVs mapped perfectly to the bacterium LF-3 reads. Most (93 %) of the bacterium LF-3 reads mapped to a single ASV assigned to an unknown genus in the family Erysipelotrichaceae (Table S2d), so ‘bacterium LF-3’ is likely a real bacterium assigned different taxonomy with other methods.

Both metagenomic approaches assigned reads to NCBI-based species-level taxonomy. However, for SRM, Bracken estimates the abundance of species based on Kraken profiles, while MEGAN-LR stops at the lowest common ancestor (LCA) assignment, so many reads are assigned to ranks above the species level (Fig. S3). Like 16S rRNA amplicon sequencing, metagenomics sequencing can characterize Archaea. For example, *Methanobrevibacter smithii* was detected with SRM and LRM through taxonomic classification and genome assembly and binning for samples from participant 5.

### Consistent taxonomic changes across methods

Clinical trial participants evaluated for this comparison displayed detectable changes in their gut microbiota from baseline to the end of investigational LBP treatment. For example, the relative abundance of the family Bacteroidaceae increased in participant 3 according to both metagenomic methods (Fig. 3b). For each sequencing method, differential abundance analysis was performed to determine whether specific bacterial families changed in abundance from baseline across individuals (Methods). The most differentially abundant families with *P*-values less than 0.2 for SRA, LRA and SRM, and *P*-values less than 0.25 for LRM are shown in Table S4. Seven families increased or decreased from baseline to treatment across two or three methods. Of these seven families, five showed the same directionality across methods, including increases in Akkermansiaceae, Bacteroidaceae, and Christensenellaceae, and decreases in Lachnospiraceae and Odoribacteraceae (Fig. 3c).

### Detection of active ingredient bacterial strain in clinical fecal samples

**Fig. 4.**
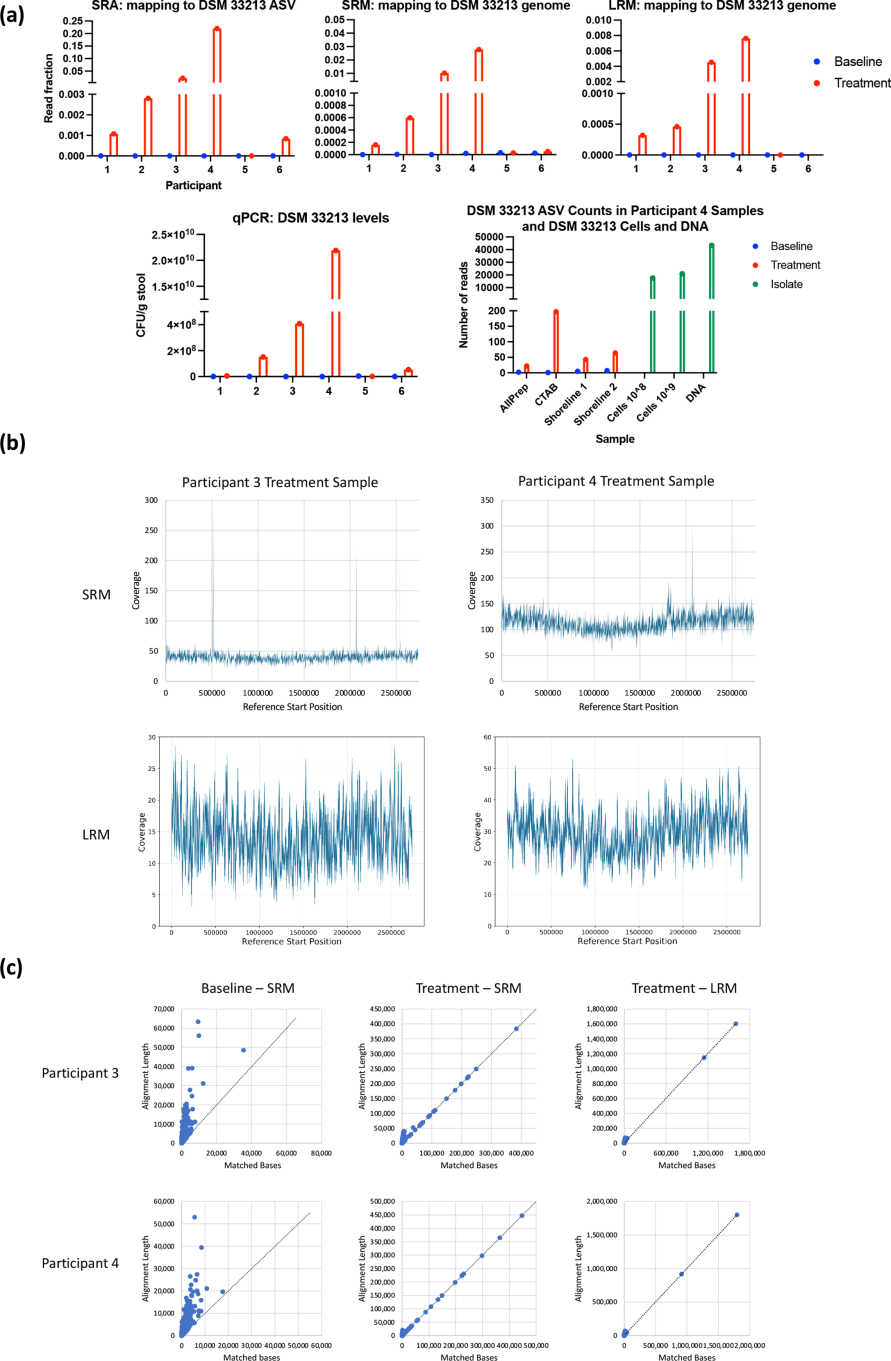
Detecting an active ingredient strain in treated samples. (a) The fraction of SRA reads in each sample at baseline or during treatment assigned to the DSM 33213 408 bp ASV (top row, left graph), or the fraction of SRM and LRM reads mapping to DSM 33213’s genome (top row, centre two graphs), compared to orthogonally verified levels of DSM 33213 in samples based on strain-specific qPCR (top right). The bottom graph shows levels of DSM 33213’s LRA ASV in participant 4’s samples at baseline and treatment. All four replicates of the sample, with DNA extracted using different methods, show DSM 33213’s ASV present in treatment but not baseline replicates. DSM 33213 cells and DNA were included in the library preparation and sequencing as positive controls and are shown in green. DSM 33213’s ASV was not detected in other participants’ treatment samples. (b) Genomic coverage plots of SRM (top) and LRM (bottom) reads from participant 3 and 4’s treatment samples. (c) Contigs assembled from SRM and LRM baseline and treatment samples from participants 3 and 4 mapped to DSM 33213’s genome. Plots show the number of matched bases versus the total length of the alignment between each contig and the DSM 33213 genome. Dotted lines have a slope of 1 to show the reference for a perfect alignment.

A key component of LBP development is the ability to track investigational active ingredient strains during and after administration. While qPCR is commonly applied to detect and quantify strains in microbial diagnostics [57], microbiome sequencing can also provide insight into the abundance of investigational LBP strains in clinical samples. To confidently detect a target bacterial strain, it must be distinguished from closely related strains that may be present in a sample. LRA analysis of DSM 33213 revealed it contains a single copy of the 16S-ITS-23S region with one mutation in the ITS region that enables separation of this strain from 29 other genomes of the same species (Fig. S4), while the strain’s 16S rRNA gene is identical to the 16S rRNA genes of nine genomes from the same species. The SRA ASV for the active ingredient strain DSM 33213 was not detected in any pretreatment baseline samples, but was detected in five out of six participants after 4 weeks of oral treatment (Fig. 4a). Two SRA ASVs assigned to the same species as DSM 33213 were detected across all samples, one matched DSM 33213’s 408 bp ASV and the other differed by four base pairs (99 % identical). This ASV was present at high abundance in participant 6’s baseline sample, and because it differed from DSM 33213’s ASV, the two strains of the same species could be differentiated (Fig. S5). The active ingredient strain’s unique 16S-ITS-23S ASV was detected in all treatment sample replicates from participant 4, but not in baseline sample replicates (Fig. 4a). The LRA ASV for DSM 33213 could not be confidently detected in other treatment samples, potentially due to the lower sequencing depth obtained for this approach.

By mapping metagenomic reads to DSM 33213’s genome, this strain was detected in most samples from treated individuals (five of six samples by SRM and four of five samples by LRM) (Fig. 4a). Abundance trends for this active ingredient strain were consistent among analysis methods, and sequencing results were consistent with trends observed with strain-specific qPCR (Fig. 4a). Levels of DSM 33213 based on SRA and SRM significantly correlated with levels based on qPCR (Pearson *r*=0.997, 0.94; *P*<0.0001, 0.01, respectively), and there was a trend towards a significant correlation between levels based on LRM and qPCR (Pearson *r*=0.84, *P*=0.07). Notably, DSM 33213 could be distinguished from a different strain of the same species present in participant 6’s baseline microbiota through metagenomic read mapping (Fig. S5).

Interestingly, in the two samples with the highest quantification of DSM 33213 (treatment samples from participants 3 and 4), mapping of LRM reads to DSM 33213’s genome generated a coverage pattern suggesting active replication, with a trough and a peak coverage profile corresponding to the bi-directional origin of replication [58] (Fig. 4b). This pattern of coverage was also clear for DSM 33213-mapped short metagenomic reads from participant 4’s treatment sample. The trough and peak pattern was less clear for participant 3’s lower coverage sample. In addition to metagenomic read coverage suggesting active replication of DSM 33213 in these two participants, assembled contigs from these two treated samples aligned with DSM 33213’s genome (Fig. 4c). Notably, the baseline samples from these two participants did not display any significant alignments to DSM 33213. From the two treatment samples, the highest quality match from the SRM contigs was a 0.45 Mbp contig with nearly 100 % of bases matching DSM 33213’s genome, and the best match from the LRM contigs was a 1.6 Mbp alignment that mapped to DSM 33213 with 99.9 % of bases matched. The top two LRM alignments belonged to a single 2.75 Mbp contig, which was itself a 98 % complete metagenome-assembled genome (MAG) assigned to the same species as DSM 33213. This demonstrates that both SRM and LRM can recover long contigs from non-endogenous strains in native microbiota, with higher mapping confidence and near-complete genomes achieved with LRM assemblies.

### Functional microbiome profiles across methods

Beyond taxonomic annotation, metagenomic sequencing assesses the functional capabilities of the gut microbiota. First, we compared results from two metagenomic sequencing pipelines optimized for either short or long reads (Methods). We decided to perform read-based analyses instead of assembly for both SRM and LRM since taxonomic profiles have been shown to be similar between raw-read assignment and assembly assignment methods, and analysis of raw-read results in more functions [59]. For SRM, read-based functional annotation was performed with HUMAnN2, which provides gene family abundances using UniProt 90 and pathway abundances with MetaCyc [40]. For LRM, reads were aligned to the NCBI nr protein database and analysed using MEGAN-LR [44]. MEGAN-LR provides annotations from multiple databases including InterPro2GO, SEED, EC and eggNOG [42].

**Fig. 5.**
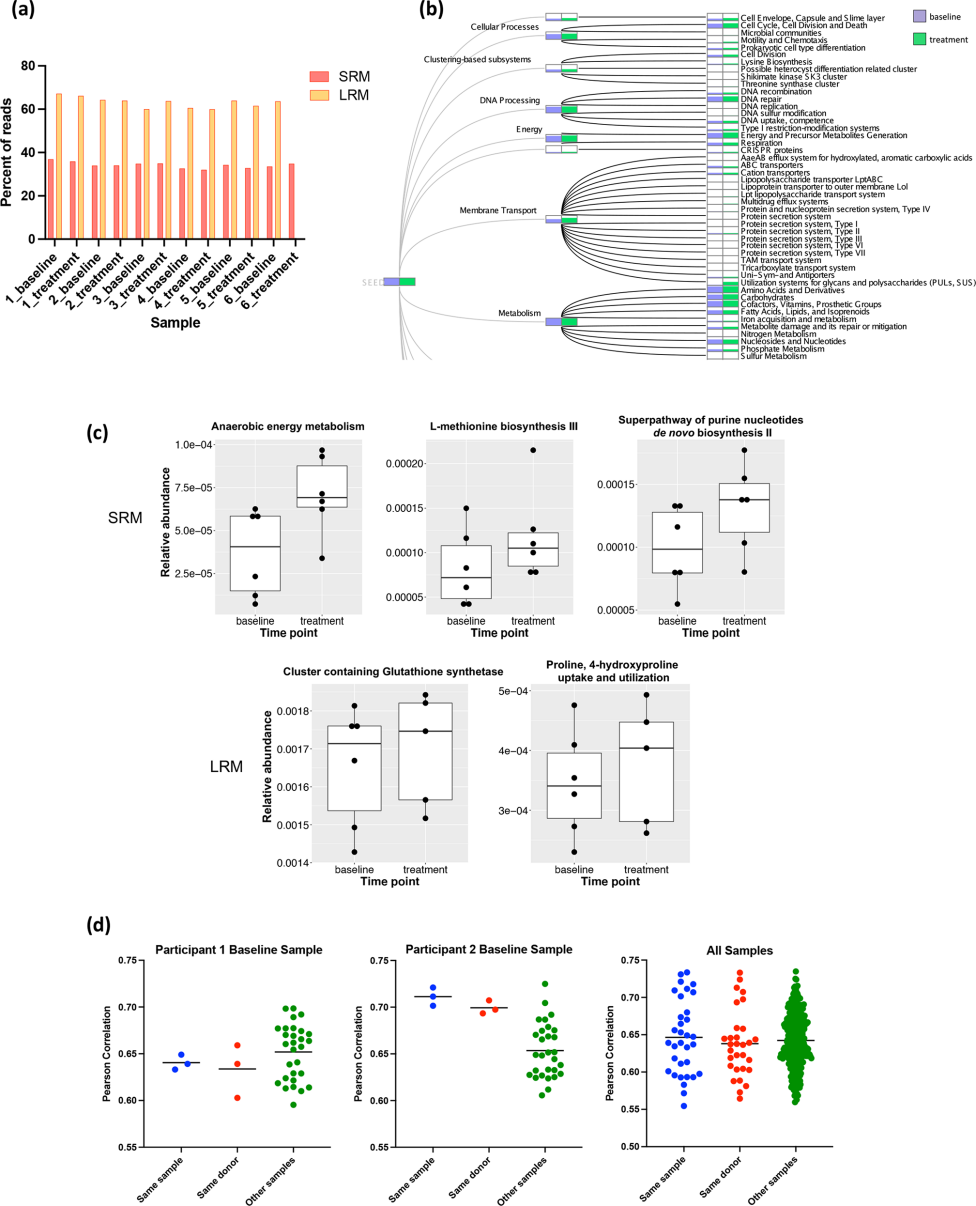
Comparison of functional annotation across methods. (a) The percent of reads for each sample with assigned, known functional annotation for SRM and LRM. Short reads were assigned functional annotations by HUMAnN2 using the UniProt90 database, and long reads were assigned functional annotations by MEGAN-LR using the SEED database. LRM library preparation failed for the treatment sample from participant 6 and was excluded from this analysis. (b) Comparing the representation of SEED-based categories from LRM analysis in participant 3’s baseline and treatment samples. (c) Significantly differentially abundant pathways comparing baseline and treatment samples based on SRM (top row) and LRM (bottom row). For SRM, pathways were annotated with MetaCyc, and for LRM, pathways were annotated with SEED. (d) Plots show the Pearson correlation between the specified sample’s LRM-based EC profile and the PICRUSt2-based EC profile based on LRA for the same sample, for the other sample from the same participant, or for all other samples. The first two plots show examples of Pearson correlations for specific samples, and the third plot shows the Pearson correlations for all samples. Horizontal lines represent the mean Pearson correlation.

To quantify the value of data from SRM and LRM pipelines, we compared the percent of samples’ reads with known functional annotations between the two pipelines. The percentage of reads with known functional annotations was substantially higher with LRM, even when limiting the comparison to annotations from one database. For all samples, an average of 34.2 % (±1.4 % sd) of reads had known functional annotations with SRM, while an average of 63.2 % (±2.4 % sd) of reads had known functional annotations for LRM (Fig. 5a). When all MEGAN-LR databases were included, 86.6 % (±3.2 % sd) of long reads were assigned known functional annotations, with an average of two to four functional annotations per read, which likely represent complete genes. To minimize differences between annotation pipelines and databases, we also assembled SRM reads into contigs and analysed them using the LRM pipeline (Methods). An average of 43.6 % (±3.7 % sd) of contigs displayed functional annotations across all databases, suggesting that the lower percentage of functional hits from SRM data is not the result of differing annotation pipelines.

The microbiota of healthy adults are relatively stable over time, but can change in response to diet, antibiotics or other perturbations [60]. This is evident in both taxonomic and functional profiles. For example, changes in the abundance of some SEED-based categories can be identified between baseline and treatment samples within a single participant (Fig. 5b). We performed differential abundance analysis between baseline and treatment samples with SRM (HUMAnN2 pathway abundances) and LRM (SEED categories). For both SRM and LRM analyses, there were pathways that significantly increased with treatment; however, the differentially abundant pathways differed between the two methods (Fig. 5c).

Unlike metagenomic sequencing, marker gene-based profiling such as 16S rRNA amplicon sequencing does not provide direct information on the gene content and functional composition of a microbial community. PICRUSt2 uses assigned taxonomy to predict the approximate functional potential of a community from marker gene sequencing profiles [36]. We performed PICRUSt2 analysis on LRA data to determine if the inferred functional profiles overlapped with those empirically determined by LRM (Methods). We systematically calculated correlations between each sample’s LRM-based Enzyme Commission (EC) profile and the LRA PICRUSt2-based EC profiles from all other samples. We observed significant correlations within and between distinct samples’ LRM EC profiles and LRA EC profiles predicted by PICRUSt2, suggesting overall conserved function in similar sample types across individuals. Surprisingly, the correlation between the LRM-based EC profile and the PICRUSt2-based profile from the same sample was not always greater than the correlation between that sample’s LRM-based EC profile and the PICRUSt2-based profiles from samples from other participants. In some cases, the mean Pearson correlation was greatest between a sample’s LRM-based EC profile and PICRUSt2-based EC profiles of samples from other participants (Fig. 5d, left). Overall, there was no significant difference between the Pearson correlations of LRM-based EC and PICRUSt2-based profiles from the same sample, from samples from the same participant, or from samples from other participants (one-way ANOVA, *P*=0.67) (Fig. 5d, right). In contrast, for LRM, EC profiles from samples from the same participant had significantly higher correlations than EC profiles from samples from different participants (unpaired t-test, P=0.02). This suggests that PICRUSt2 cannot fully recapitulate metagenomics-based functional metabolic profiles.

### Diverse MAGs were recovered from metagenomic assemblies

**Fig. 6.**
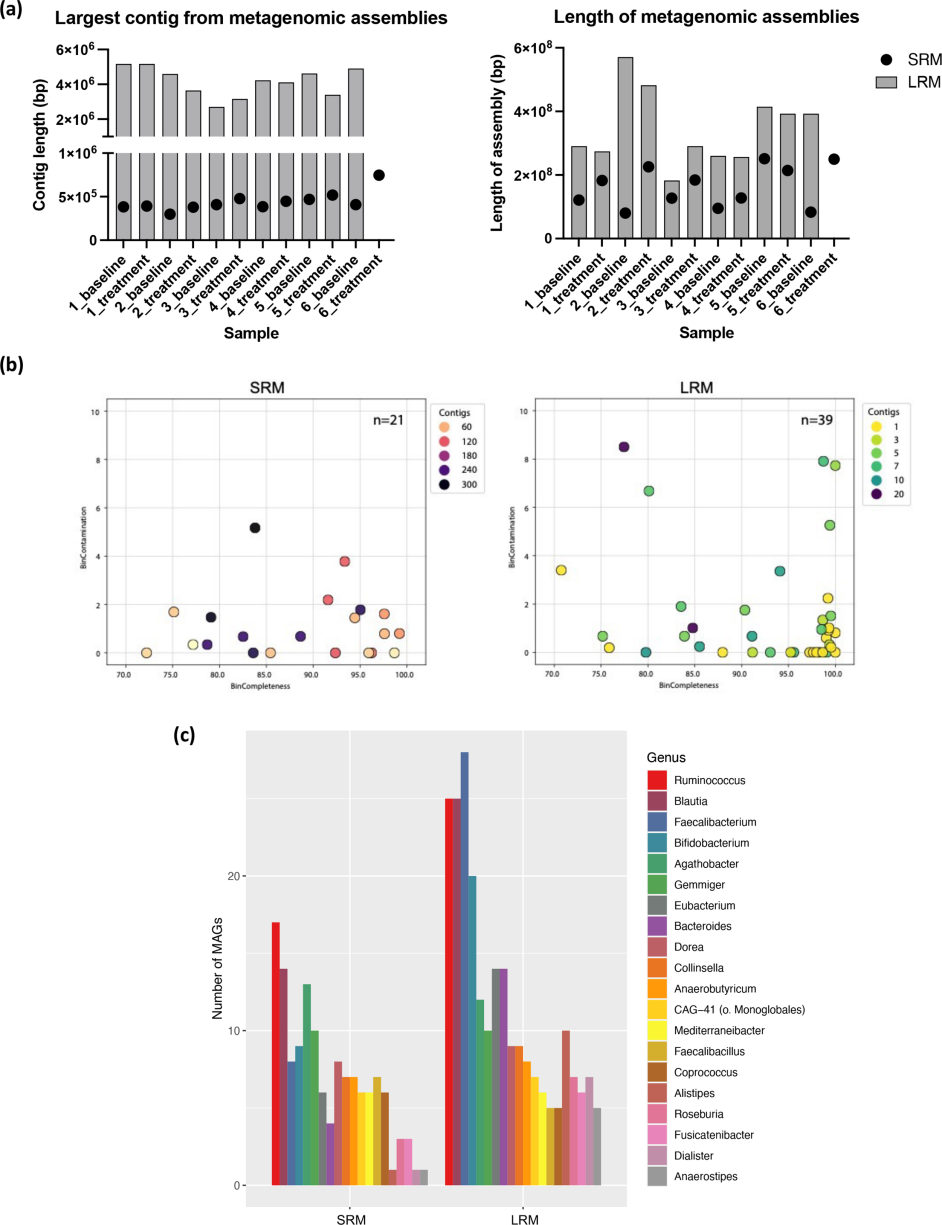
Comparison of metagenomic assemblies and metagenome assembled genomes (MAGs) for SRM and LRM. (a) Length of the longest contig in bp from each sample’s metagenomic assemblies from SRM and LRM (left). Total length of each sample’s metagenomic assemblies for SRM and LRM (right). (b) MAGs passing filtering criteria from participant 5’s baseline sample from SRM (left) and LRM (right). (c) The number of MAGs passing filtering from all samples for the 20 genera with the greatest number of recovered MAGs, for SRM (left) and LRM (right).

One of the major benefits of metagenomic sequencing is the potential to recover microbial genomes that may not exist in genome databases. Reads from SRM and LRM were assembled into contigs using MetaSPAdes and hifiasm-meta, respectively. Assemblies from LRM were substantially more complete than assemblies from SRM. The average N50 for SRM assemblies was just under 7 kb, whereas the average N50 for LRM assemblies was 164 kb (Table S5). There was an average of 69 999 contigs per assembly from SRM and 8091 contigs per assembly from LRM. The average largest contig per sample was 0.44 Mbp for SRM assemblies and 4.2 Mbp for LRM assemblies (Fig. 6a), and the LRM total assembly lengths were on average double the size of the SRM assembly lengths (346 million versus 161 million bases; Fig. 6a, Table S5). The two exceptions, where the total assembly length was much shorter for SRM, were the baseline samples from participants 2 and 6. These two samples had the fewest number of short metagenomic reads (12.4 million and 23.6 million paired reads, respectively), highlighting the need for greater sequencing depth for SRM if metagenomic assembly is desired. The average GC content of SRM and LRM assemblies were similar (45.9 and 46.0 %, respectively), suggesting that there were no drastic compositional differences between the two datasets.

For both SRM and LRM, genome binning and evaluation was performed using PacBio’s HiFi-MAG-Pipeline, and genome bins were filtered to generate high-quality metagenome assembled genomes (MAGs). To generate MAGs from SRM data, the filtering criteria were relaxed for SRM to accept MAGs of lower quality with 500 or fewer contigs instead of 20 or fewer contigs as specified in the original LRM pipeline. Even using the relaxed filtering criteria for SRM, there were about twice as many high-quality MAGs derived from LRM: an average of 33 high-quality MAGs per sample compared to 17 MAGs for SRM (Table S5). Across all samples there were 367 total MAGs from LRM and 206 total MAGs from SRM. MAGs from LRM were more complete than MAGs from SRM, with an average of 4 contigs per MAG for LRM versus 138 contigs per MAG for SRM. For participant 5’s baseline sample, there were 21 and 39 MAGs passing filtering from SRM and LRM, respectively (Fig. 6b). For this sample, there were only two SRM MAGs that were more than 98 % complete, while there were 18 LRM MAGs that were more than 98 % complete. Moreover, 8 of the >98 % complete LRM MAGs consisted of a single contig, representing complete or near-complete bacterial genomes. There was only one SRM MAG containing fewer than 20 contigs, while all the LRM high-quality MAGs by definition contained fewer than 20 contigs.

The MAGs recovered from SRM and LRM encompassed a diverse range of taxa, and there was a high degree of overlap between methods. For example, for participant 5’s baseline sample, there were 11 MAGs across both sequencing methods assigned the same species-level GTDB taxonomy (Table S6). There were 62 and 89 unique genera recovered from all samples with SRM and LRM, respectively. The five genera with the highest number of MAGs across both methods were *Ruminococcus* (42 MAGs), *Blautia (*39), *Faecalibacterium* (36), *Bifidobacterium* (29) and *Agathobacter* (25). Fig. 6 shows the top 20 genera with the greatest number of MAGs recovered from both methods.

## Discussion

The number of microbiome-based therapies in preclinical and clinical development has grown tremendously over the last decade, and now include diverse approaches such as faecal microbiota transplants, single bacterial strains, bacterial consortia and genetically modified strains [61]. Although diverse in their approaches, these programmes overlap in their need to accurately characterize temporal microbiome dynamics in clinical trial participants. While studies of the human microbiome have largely relied on highly accurate short-read Illumina DNA sequencing, the introduction of long-read sequencing justifies a re-examination of these approaches. The superior taxonomic resolution of long-read full-length 16S amplicon sequencing has been well described [16], however these comparisons are limited to amplicon-based methods and nonclinical datasets.

The overall proportions of phyla and families observed were similar across the four methods applied in this comparison, suggesting that these methods are equally suited to generate lower-resolution taxonomic profiles. A notable difference was a greater proportion of ‘unknown phyla’ observed with the LRA approach due to reads annotated as bacterium LF-3. This human gut isolate is not classified at higher taxonomic levels in NCBI (Accession No. PRJEB6481), but in GTDB it is classified as the species *Faecalibacillus intestinali*, in the phylum Firmicutes and family Erysipelatoclostridiaceae. SRA ASVs mapped to many LRA reads assigned to bacterium LF-3 across multiple participants (Table S2b) with Greengenes-assigned taxonomy was an unknown species in the family Erysipelotrichaceae. SRA ASVs mapped to many LRA reads assigned to bacterium LF-3 across multiple participants (Table S2b). The Greengenes taxonomy assigned to these ASVs was an unknown species in the family Erysipelotrichaceae. Notably, the overall taxonomic profiles of the samples for LRA would more closely resemble other methods at the phylum level using a different default reference database. These differences highlight the significant impact of reference databases on outputs from these methods.

Other notable differences in taxonomic profiles included a significantly greater proportion of Verrucomicrobia with both short-read methods and a greater proportion of Actinobacteria with SRM. The Verrucomicrobia genus *Akkermansia* has been shown to be overrepresented in 16S rRNA analysis with V3-V4 primers [62]. Accurate representation of Bifidobacteria (Actinobacteria) depends on thorough mechanical lysis during DNA extraction [63], so the lower representation of Bifidobacteria in the LRA and LRM taxonomic profiles could be due in part to differences in efficiency of DNA extraction. 16S rRNA analysis based on the V3-V4 region and other regions has also been shown to underestimate the abundance of Bifidobacteria [64].

Differences in taxonomic profiles across methods could be due to a variety of factors aside from database differences and DNA extraction methods. For example, an average of just 24 % of SRA reads mapped to LRA reads from the same sample (Table S2a). This could be due to lower read coverage with LRA, chimaeras from PCR amplification, or sequencing errors from both methods. Also, some SRA ASVs mapped perfectly to multiple LRA reads, sometimes with different taxonomic assignments. For example, for participant 1’s baseline sample, one SRA ASV mapped to many LRA reads assigned to the genus *Megamonas *, but this ASV also mapped to LRA reads assigned to more distantly related taxa including the genera *Escherichia* and *Bifidobacterium* (bold in Table S2b). Despite these differences in taxonomic assignments, all four methods preserved relationships between the samples (Fig. 2b).

Taxonomic resolution is an important factor to consider when choosing a sequencing method, as increasing evidence shows that strains are the functional units of the microbiota [57]. For example, although the species *Cutibacterium acnes* is a dominant skin commensal, the distribution of *C. acnes* strains significantly differs between acne patients and healthy individuals [58]. Metagenomic sequencing typically provides greater taxonomic resolution than 16S rRNA sequencing, and in our analysis both LRM and SRM generated species-level taxonomic profiling. For SRA, the fraction of reads assigned to species was considerably higher when the Greengenes taxonomy database was used instead of the silva taxonomy database, again highlighting the significant impact of the chosen reference database. LRA, unlike all other methods, provided strain-level resolution for an average of 13.4 % of reads across all samples. In addition, LRA provided the greatest resolution in identifying the highest number of unique taxa per sample.

Tracking active ingredient strains is essential for evaluating their fate, including engraftment or clearance, after administration. All four microbiome analysis methods applied here could detect a target investigational LBP strain in treated samples and relative levels of this strain were comparable across methods. LRA analysis showed that the active ingredient strain DSM 33213’s ASV contains a single mutation that enables its distinction from all other known genomes of the same species. In this analysis, all methods were able to distinguish DSM 33213 from closely related strains, an interesting finding given the high conservation of the V3-V4 region in most strains of the same species. Although LRA had high specificity in detecting DSM 33213, its sensitivity was lower. For high strain sensitivity with LRA, higher read coverage is likely necessary. Overall, LRA is a cost-effective method for tracking closely related strains in microbiota samples without the need for costly metagenomic sequencing, as evidenced in the tracking of closely related strains in the faecal microbiomes of premature infants [20]. Due to its higher sensitivity, qPCR should remain the gold standard for detecting specific strains. Although sequencing can likely only detect strains above a certain threshold of abundance in the gut, it can provide additional information on higher abundance target strains. For example, here we show that metagenomic approaches may also provide information on the replication activity of an active ingredient strain from stool DNA. SRM and LRM reads mapping to DSM 33213’s genome generated similar peak-and-trough coverage patterns, suggestive of an actively replicating strain. Assembling reads into contigs further increases mapping confidence; both SRM and LRM resulted in high confidence matches between contigs and an active ingredient strain genome in treated samples, but not in baseline pretreatment samples. The longest alignments for SRM contigs were less than 500 kb, while the longest alignments for LRM contigs were over 1.6 Mbp, increasing confidence in active ingredient strain detection with the LRM.

Since metagenomic sequencing provides direct information about microbial gene content, it can help elucidate the mechanism of the microbiota’s contribution to health or disease or the potential mechanism of action of an LBP. Comparing genes encoded by the microbiome across methods, the percentage of reads with known functional annotations was substantially higher with LRM compared to SRM (63 versus 34 %). Even though the cost of LRM is higher, the amount of useful data is substantially greater and requires less bioinformatic processing since HiFi reads are high-quality consensus reads; in our study they displayed a median quality score of Q40 (99.99 % accuracy). Long reads contain multiple genes in their original genomic context, increasing confidence in their taxonomic and functional annotations. There was no substantial overlap in differentially abundant pathways across methods, which could be attributed to the limited number of samples used in this analysis, differences in reference databases and the smaller fraction of data with known annotations for SRM. Even though the amount of raw sequencing data per sample was comparable between SRM and LRM (~10 Gb), LRM resulted in more assembled contigs and about twice the number of high-quality MAGs per sample, representing near-complete genomes produced from complex microbial communities. Although the SRM MAGs were significantly more fragmented, there was overlap in the taxonomic composition between SRM and LRM MAGs.

A notable challenge in comparing sequencing methods is that each method requires analysis pipelines and databases that are optimized for the distinct nature of the raw sequencing data. The impact of chosen databases was especially obvious when different databases were applied to the same sequencing method. For example, using the Greengenes database to annotate SRA ASVs increased the fraction of reads assigned to species compared to the silva database. To overcome the limitations of comparing outcomes generated using different reference databases, we directly mapped SRA ASVs to LRA long reads and assessed the overlap in inferred communities and compared taxonomic resolution. For functional annotation of metagenomic data, independent of the analysis method, the proportion of data with known functional annotations was substantially higher with LRM than SRM. To overcome pipeline differences for this analysis, contigs assembled from SRM were analysed using the LRM functional profiling pipeline. Even when the same pipeline was used, the proportion of assembled SRM contigs with functional annotations was still lower than the proportion of LRM reads with functional annotations.

One limitation of this study is the lack of a positive control across all methods, such as a microbial community with known abundances, that could serve as a ground truth for taxonomic composition. Negative controls were included but resulted in a negligible number of reads and were excluded from analyses. Sequencing and analysis of high-biomass samples such as stool are typically not compromised by contaminating DNA in extraction, amplification and library-preparation kits [59]. A notable exclusion of this study is the use of nanopore long-read sequencing technology (Oxford Nanopore). Nanopore sequencing is attractive due to its low cost, portability and rapid data generation, and Oxford Nanopore’s MinION has been leveraged for full-length 16S amplicon sequencing of microbial communities [65], in the sequencing and construction of MAGs from human stool DNA [60], and can produce very long reads with higher species-level resolution when compared to Illumina short reads [61]. However, despite improvements in computational error correction [66], the comparatively low throughput and low nucleotide accuracy of nanopore sequencing make it a less suitable for clinical trial microbiome sequencing.

Microbiome scientists and LBP developers must weigh the pros and cons of available approaches to answer their core research questions. This comparison of four microbiome sequencing and analysis approaches highlights the overlapping and distinct value of SRA, LRA, SRM and LRM approaches. Overall, taxonomic profiles overlapped across these methods, supporting their application for low-resolution taxonomic profiling of microbial communities. The increasing importance of species and strain-level resolution in microbiome studies warrants the application of newer approaches, such as LRA sequencing, shown here to generate high-confidence species and strain-level profiles. This study also shows that detection of an investigational LBP strain in clinical trial participants can be achieved with diverse methods; however, confident strain tracking using SRA depended on an absence of closely related taxa. Studies investigating the encoded functional capacity of the microbiome should prioritize metagenomic approaches (LRM/SRM). We also show that metagenomic approaches can provide added value in the estimation of strain growth rates and the discovery of novel metagenomes. Metagenomic assemblies of short and long reads were compositionally similar, although long-read assemblies were more complete. LRM was superior in the recovery of metagenomes with a comparable amount of sequence data. Overall, long-read approaches were superior to short-read approaches in the confidence of associated taxonomic and functional annotations, however the detection of low-abundance features using long-read approaches at standard sequencing depths was limited compared to comparable short-read methods. Researchers must weigh the importance of detecting low abundant features (sensitivity) with the need for higher resolution and confidence in annotations (specificity). The strong impact of the choice or default reference database applied in these analyses highlights the importance of performing parallel analyses with multiple reference databases regardless of sequencing approach.

## Supplementary Data

Supplementary material 1

Supplementary material 2
