# Supplementary material for: Finding the right fit: evaluation of short-read and long-read sequencing approaches to maximize the utility of clinical microbiome data
Source: Microb Genom. 2022 Mar 18;8(3):000794. doi: 10.1099/mgen.0.000794 (PMC9176275; doi:10.1099/mgen.0.000794)
Supplement: Supplementary material 1 [file mgen-8-0794-s001.pdf]

Supplementary Figures

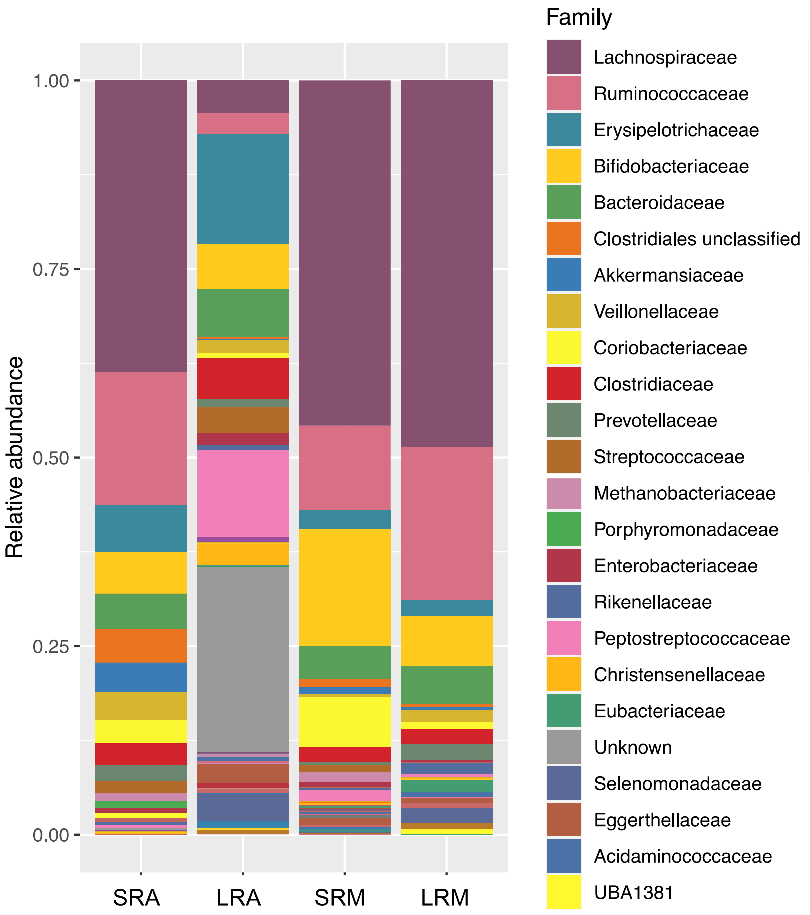

Fig. S1. Taxonomic comparison across methods with different databases. Taxonomic comparisons across methods at the family level. Bar graphs show the relative abundance of each family across all samples. The 24 most abundant families across all methods are shown. For SRM, an older version of the NCBI database was used to assign taxonomy, while a more recent NCBI database was used in Fig. 2A. For LRM, taxonomy was assigned using GTDB.

A.

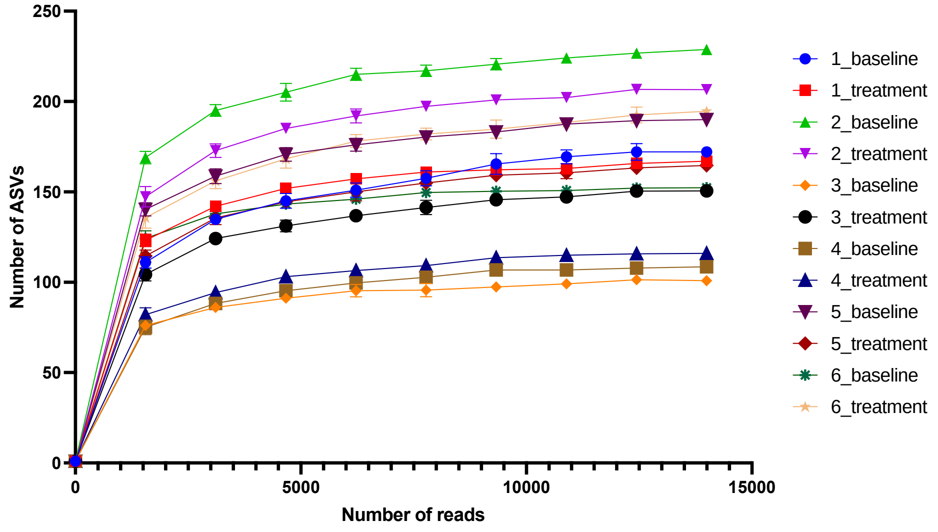

B.

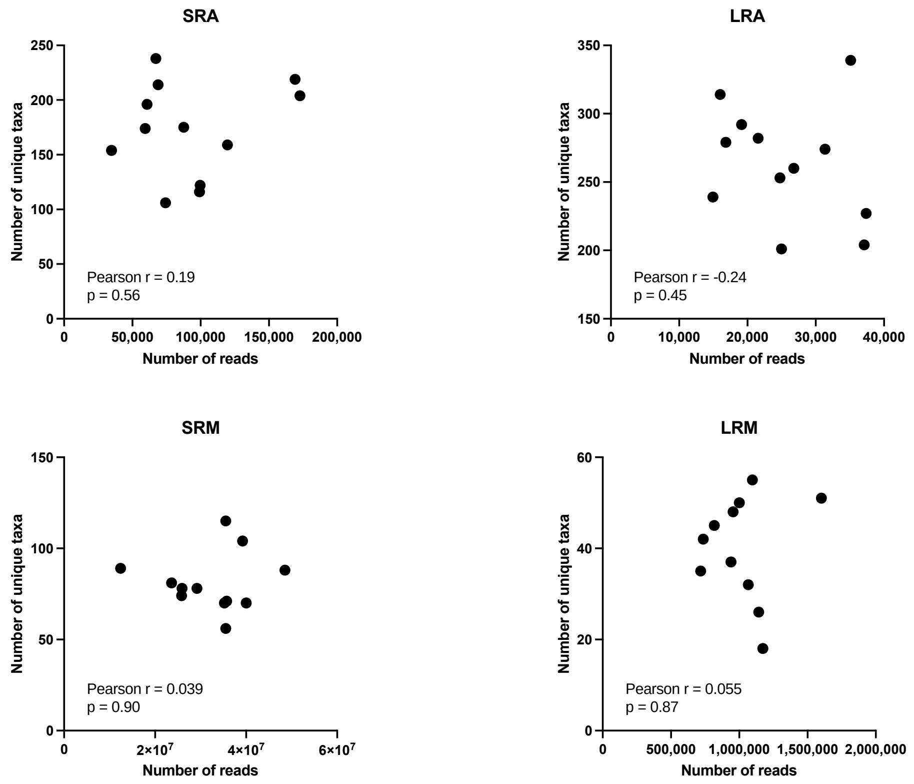

Fig. S2. (A) Rarefaction curve showing the number of ASVs detected in the twelve stool samples at various sequencing depths with SRA. **(B)** Scatterplots for all four sequencing methods show the number of reads sequenced versus the number of unique taxa detected for each sample. None of the Pearson correlations are significant.

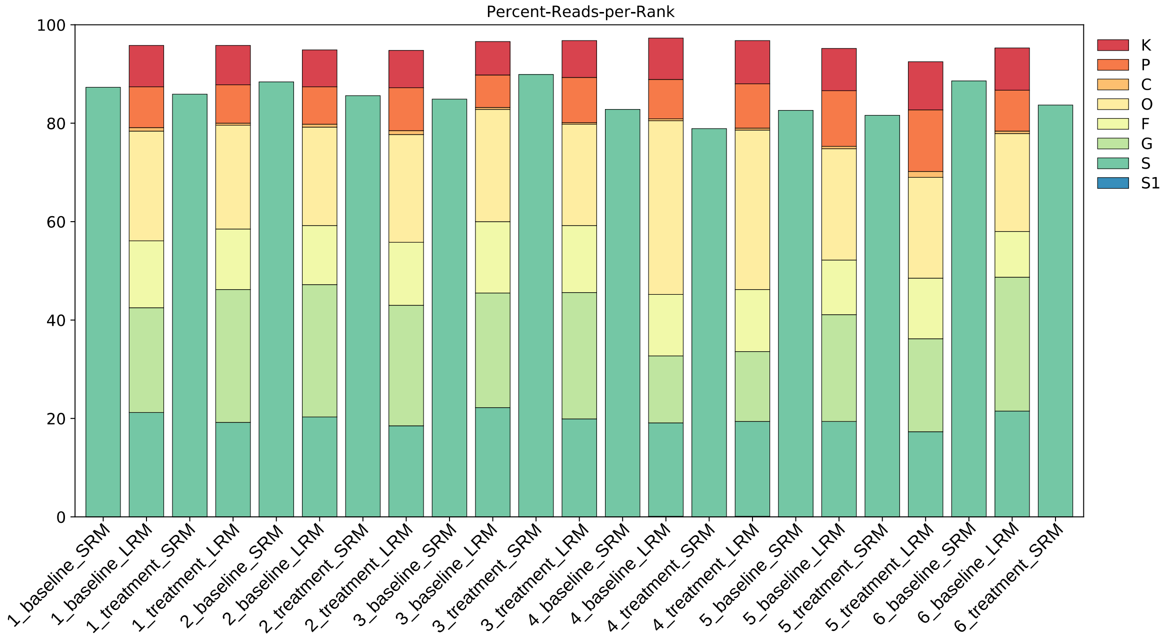

Fig. S3. Percent of reads assigned to taxonomic rank for SRM and LRM. The percent of metagenomic reads assigned to kingdom (K), phylum (P), class (C), order (O), family (F), genus (G), species (S) or strain (S1), shown by sample for both SRM (e.g., 1_baseline_SRM) and LRM (e.g., 1_baseline_LRM). Taxa below 0.05% relative abundance were excluded for both methods.

A.

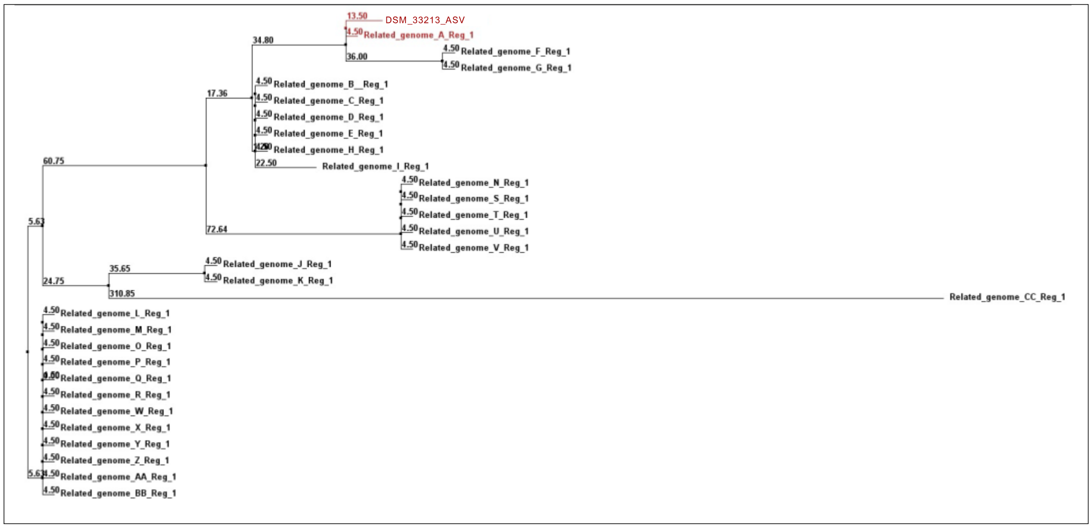

B.

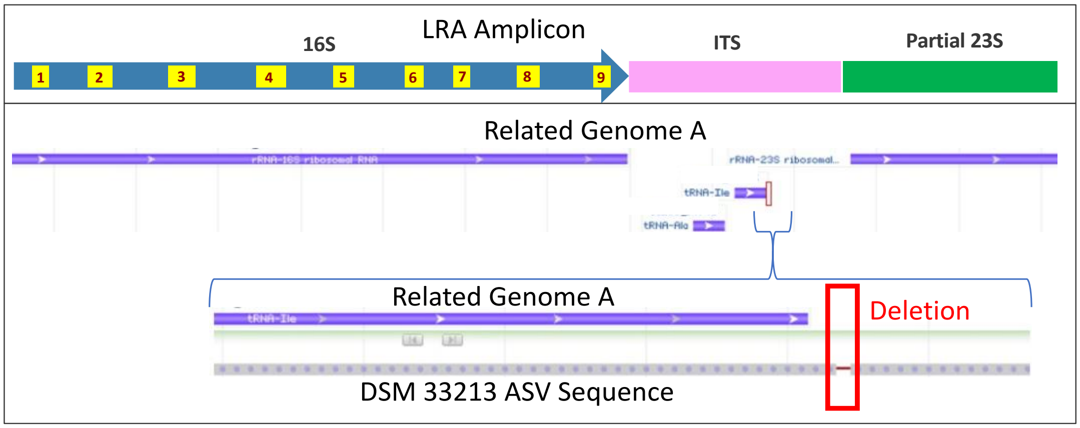

Fig. S4. Comparison of DSM 33213's ASV to closely related genomes. (A) LRA phylogenetic tree based on alignment of DSM 33213's ASV to the 29 most closely related genomes from NCBI. DNA alignments in Jalview were used to calculate a Neighbor Joining tree. DSM 33213's ASV and the most closely related sequence (Related_genome_A) from NCBI are shown in red text. **(B)** DSM 33213's 2534 bp ASV was aligned with the top hit of the 29 closely related genomes in NCBI. The deletion site in DSM 33213's ASV sequence is in a non-coding site just outside the tRNA Ile gene in the ITS region, denoted with a red box. Related Genome A was 2536 bases long and contained an insertion in the same non-coding region between the tRNA Ile and 23S gene, closer to the 23S gene. All other LRA sequences for closely related genomes were 2535 bases long and differed from DSM 33213's ASV with multiple base substitutions (not shown).

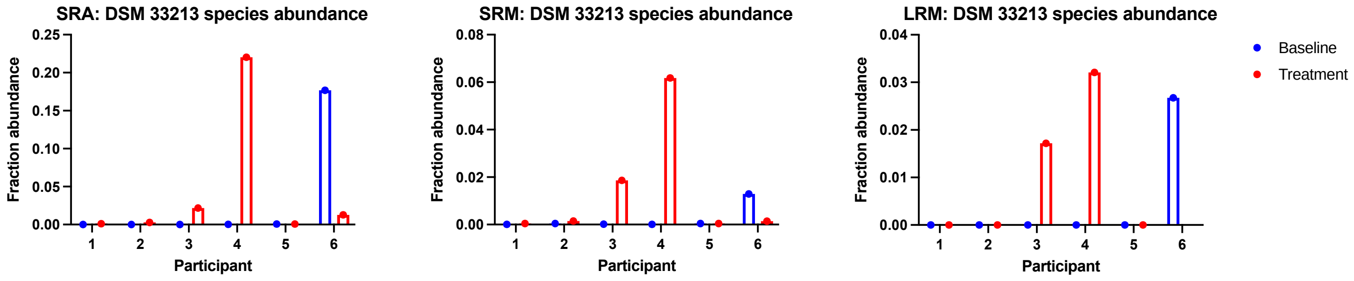

Fig. S5. Relative abundance of the species to which DSM 33213 belongs across participants and methods. For SRA, there were two ASVs assigned to the species, one matching DSM 33213's ASV and another distinct ASV. The relative abundances of these two ASVs were summed.
